# Hormonal Crosstalk Redefines Iron Deficiency Morphophysiological Responses: Insights From Tomato Auxin‐ and Ethylene‐Related Double Mutants

**DOI:** 10.1111/ppl.71181

**Published:** 2026-11-06

**Authors:** Carolina Cristina Monteiro, Alexandre Virgínio de Melo, Evilásio Anísio Costa Filho, Ricardo Antunes Azevedo, Tiago Tezotto, Renato de Mello Prado, Marina Alves Gavassi, Frederico Rocha Rodrigues Alves, Priscila Lupino Gratão, Rogério Falleiros Carvalho

**Affiliations:** ^1^ School of Agricultural and Veterinary Sciences, Department of Biology São Paulo State University (UNESP) Jaboticabal SP Brazil; ^2^ Center of Exact and Natural Sciences, Department of Systematics and Ecology, Laboratory of Plant Physiology and Evolution Federal University of Paraíba (UFPB) João Pessoa PB Brazil; ^3^ Laboratory for Evaluation and Development of Biomaterials of the Northeast (CERTBIO) Federal University of Campina Grande (UFCG) Campina Grande PB Brazil; ^4^ Luiz de Queiroz College of Agriculture (ESALQ), Department of Genetics University of São Paulo (USP) Piracicaba SP Brazil; ^5^ Luiz de Queiroz College of Agriculture (ESALQ), Department of Soil Science University of São Paulo (USP) Piracicaba SP Brazil; ^6^ School of Agricultural and Veterinary Sciences, Department of Soil Science São Paulo State University (UNESP) Jaboticabal SP Brazil; ^7^ School of Sciences, Department of Biological Sciences São Paulo State University (UNESP) Bauru SP Brazil

**Keywords:** *diageotropica*, *epinastic*, Fe homeostasis, *Never ripe*, *Solanum lycopersicum*

## Abstract

Plants have evolved complex strategies for iron (Fe) acquisition, yet the coordination of underlying hormonal mechanisms remains elusive. This study investigated how auxin and ethylene signaling interact to regulate Fe‐deficiency responses using single (auxin‐insensitive *diageotropica*, *dgt*, ethylene‐insensitive *Never ripe*, *Nr*, ethylene‐overproducer *epinastic*, *epi*) and double (*dgt/Nr*, *dgt/epi*) hormonal mutants of tomato ( *Solanum lycopersicum* cv. Micro‐Tom). We analyzed growth parameters, photosynthetic pigments, oxidative stress markers, antioxidant enzyme activities, nutrient profiles, and the expression of Fe acquisition genes. Results revealed that ethylene strongly contributes to the transcriptional regulation of key Fe acquisition genes, as *epi* and *dgt/epi* mutants exhibited strong upregulation of these genes under Fe deficiency, indicating that enhanced ethylene biosynthesis up‐regulates Fe acquisition genes even with impaired auxin sensitivity. Conversely, root and shoot development were primarily constrained by auxin sensitivity. The *dgt* allele showed clear dominance over *Nr* and *epi*, as all *dgt*‐harboring genotypes displayed significantly reduced root systems and leaf area regardless of Fe availability. Furthermore, Fe deficiency decreased the activities of antioxidant enzymes, increasing oxidative stress and altering mineral distribution, notably through enhanced Mn accumulation in leaves and Cu in roots. Our findings demonstrate that while ethylene is a central regulator of the Fe‐deficiency transcriptional response, auxin sets the structural framework for root adaptation.

## 1 Introduction

Iron (Fe) is an essential micronutrient for plants, primarily due to its role in redox reactions as a cofactor of proteins involved in the electron transport chains in chloroplasts and mitochondria, as well as in chlorophyll biosynthesis and nutrient assimilation processes (Przybyla‐Toscano et al. 2021). Fe bioavailability to plants is limited depending on soil pH and redox status, as Fe^3+^ readily precipitates as insoluble oxides under alkaline conditions (Marschner 2012). As a major limiting factor for plant growth and productivity, Fe deficiency results in leaf chlorosis, reduced biomass accumulation, and increased susceptibility to oxidative stress, given that many ROS‐detoxifying enzymes, such as catalase (CAT) and ascorbate peroxidase (APX), rely on Fe as a cofactor for their catalytic activities (Tripathi et al. 2018; Vélez‐Bermúdez and Schmidt 2023).

To cope with Fe deficiency, plants have evolved diverse and complex adaptive mechanisms to optimize Fe acquisition and maintain Fe homeostasis (Zhang et al. 2019). Eudicotyledonous species, such as tomato, enhance the activity of the plasma membrane FERRIC REDUCTASE OXIDASE 1 (FRO1) in roots, reducing Fe^3+^ to Fe^2+^ (Robinson et al. 1999; Li et al. 2004) and increasing Fe^2+^ uptake capacity through higher expression of the Fe^2+^ transporter *IRT1* (Korshunova et al. 1999). Root morphology is also altered in response to Fe deficiency. Plants exhibit a shallower architecture due to the inhibition of primary root elongation, coupled with the promotion of subapical root hairs and cluster roots to increase the soil contact surface and Fe mobilization through chemical modification of the rhizosphere (Jin et al. 2008).

Fe deficiency also leads to marked changes in the nutritional profile of plants, particularly affecting the uptake of other divalent cations (Connorton et al. 2017). This occurs mainly because key components of the Fe uptake machinery, such as the Fe^2+^ transporter IRT1, exhibit low substrate specificity and can transport other divalent metals, including Mn^2+^, Zn^2+^, Cu^2+^ and Ni^2+^ (Korshunova et al. 1999; Nishida et al. 2011; Rai et al. 2021). As a consequence, −Fe plants often show increased accumulation of other metals in roots and shoots, which may partially compensate for redox functions but can also result in ionic imbalance and toxicity under certain conditions (Vert et al. 2002; Kanai et al. 2009; Lešková et al. 2022). Therefore, Fe deficiency not only disrupts Fe homeostasis but also broadly impacts mineral nutrition, highlighting the tight interconnection between Fe acquisition strategies and the regulation of other micronutrients.

Limited Fe availability compromises ATP production and promotes redox imbalances due to the dysfunction of cytochromes, ferredoxins, and other Fe‐dependent proteins, leading to the accumulation of reactive oxygen species (ROS) (Vigani 2012). Under these conditions, root system plasticity emerges as an adaptive yet energetically demanding strategy to enhance soil exploration and Fe acquisition (Jin et al. 2008; Higa et al. 2010). Root growth remodeling under Fe deficiency is tightly regulated by hormonal signaling, particularly involving auxin and ethylene, which act in an integrated manner. Auxin contributes to the reprogramming of root growth by promoting cell elongation and lateral root formation, whereas ethylene modulates both auxin sensitivity and the activation of Fe deficiency responses, including changes in redox metabolism and the expression of genes involved in Fe uptake, such as *FRO1* and *IRT1* (Lucena et al. 2006, 2019). Furthermore, auxin and ethylene work synergistically in a hormonal crosstalk, wherein auxin promotes ethylene biosynthesis, while ethylene, in addition to stimulating auxin synthesis, modulates auxin transport across distinct root tissue zones (Lucena et al. 2015; Romera et al. 2011; Vaseva et al. 2021). Thus, the interplay among energy status, redox balance, and hormonal signaling constitutes a central regulatory axis in root adaptation to Fe deficiency. Unraveling the complex hormonal interactions and crosstalk underlying these adaptive morphophysiological responses can also support the development of new biotechnological strategies and tools to enhance nutrient use efficiency and the breeding of tolerant cultivars to thrive under Fe‐limiting conditions (Du et al. 2023).

Tomato provides a powerful model for dissecting hormonal regulation due to its well‐characterized mutants. The *diageotropica* (*dgt*) mutant shows low sensitivity to auxin due to a defect in a cyclophilin biosynthesis gene, a putative component of auxin signal transduction (Oh et al. 2006). The *Never ripe* (*Nr*) mutant exhibits low sensitivity to ethylene, having a defective ethylene receptor (Wilkinson et al. 1995), while the *epinastic* (*epi*) overproduces ethylene, behaving as a constitutive ethylene‐response mutant, but the causal gene has not yet been described (Fujino et al. 1988; Barry et al. 2001). In addition, the possibility of cross−fertilization and generation of double mutants offers an opportunity to assess interactions between auxin and ethylene pathways, revealing hierarchical or organ‐specific dominance between hormonal signals. While these mutants have been instrumental in studies of development and ripening (Carvalho et al. 2011), their combined effects on nutrient stress responses can also be explored to characterize the complex hormonal crosstalk.

While the molecular interplay between auxin and ethylene is well‐established in modulating plant development, whether one hormone holds primary control over morphological adaptations under Fe deficiency or whether they act as co‐equal signals remains an open question. Therefore, the present study investigates how auxin‐ and ethylene‐related mutations, individually and in combination, influence morphological, physiological, biochemical, nutritional, and transcriptional responses to Fe deficiency in tomato. By integrating analyses of growth traits, chloroplast pigments, oxidative stress markers, antioxidant enzyme activity, mineral nutrient accumulation, and the expression of important Fe acquisition genes, we aim to investigate how interactions between hormonal pathways modulate Fe deficiency acclimation. Our findings provide new insights into the hierarchical roles of auxin and ethylene in regulating Fe homeostasis and reveal how hormonal crosstalk shapes multi‐level stress responses in tomato.

## 2 Materials and Methods

### 2.1 Generation of Tomato Hormonal Mutants and Experimental Growth Conditions

Wild‐type (WT) *Solanum lycopersicum* L. cv. “Micro‐Tom” and near‐isogenic lines harboring the hormonal signaling mutations *diageotropica* (*dgt*), *Never ripe* (*Nr*), and *epinastic* (*epi*) introgressed into the Micro‐Tom background were used (Fujino et al. 1988; Wilkinson et al. 1995; Oh et al. 2006; Carvalho et al. 2011). Double mutants *dgt/Nr* and *dgt/epi* were generated by crossing every single mutant (*Nr* or *epi*) with *dgt*, using *dgt* as the pollen donor. F_1_ plants were self−fertilized and homozygous double mutants were identified in the F_2_ generation based on their distinct phenotypic markers: delayed fruit ripening (yellowish fruits) for *Nr*‐bearing mutants and a pointed fruit apex for *epi*‐bearing mutants. *dgt* mutation was further confirmed in single and double mutants by PCR and DNA sequencing as *dgt* ^*G137R*^ (*dgt* ^*1‐1*^; Oh et al. 2006). Homozygous individuals were confirmed by non‐segregating F_3_ progeny testing.

Seeds were sown in a 1:1 (v/v) mixture of Plantmax HT (Eucatex) and vermiculite supplemented with 1 g L^−1^ NPK (10–10–10) and 4 g L^−1^ dolomitic limestone. Seedlings were grown under greenhouse conditions until 20 days of age, after which they were transferred to a nutrient solution containing: 1.25 mM Ca(NO_3_)_2_, 1.25 mM KNO_3_, 0.5 mM MgSO_4_, 0.25 mM KH_2_PO_4_, 10 μM H_3_BO_3_, 1 μM MnCl_2_, 0.5 μM ZnSO_4_, 0.5 μM CuSO_4_, 0.05 μM H_2_MoO_4_, and 20 μM Na_2_Fe‐EDTA, adjusted to pH 5.8 with KOH.

Plants were maintained under controlled conditions (12 h photoperiod, 25°C ± 3°C) for 20 days and then transferred either to a nutrient‐sufficient solution (+Fe) or to a Fe‐deficient nutrient solution (−Fe). Plants were harvested and analyzed 4 days after treatment for gene expression analysis, to capture early transcriptional reprogramming prior to confounding phenotypic changes, and after 24 days for all remaining variables.

### 2.2 Morphological Analyses

Primary root length and lateral root density were assessed after staining roots with 10 g L^−1^ methylene blue for 3 min. Root images were acquired using an optical scanner (Hewlett Packard) and analyzed with the Delta‐T Image Analysis System (Delta‐T Devices). Leaf area was obtained by scanning individual leaves and analyzing images using QUANT software (Vale et al. 2003). Dry mass was determined after drying samples at 65°C for 96 h, using an analytical balance (±0.0001 g; Denver Instrument AA‐200).

### 2.3 Nutrient Content Quantification

Nutrient concentration was determined following Chilimba et al. (2011). Dried tissue samples of approximately 100 mg were digested in 3 mL of 70% ultrapure HNO_3_ in a microwave digestion system (Multiwave 3000, Anton Paar) at 20 bar for 45 min. Samples were diluted in ultrapure water and analyzed using a inductively coupled plasma optical emission spectrometer (ICP‐OES) (JobinYvon, JY50P).

### 2.4 Chlorophyll, Carotenoid and Biochemical Stress Markers Quantification

For chlorophyll and carotenoid quantification, two 10 mm diameter fresh leaf discs were cut and pigments were extracted in 100% acetone for 24 h at 4°C in the dark. Absorbance at 662, 645, and 470 nm was measured with a UV–Vis spectrophotometer (PerkinElmer). Pigments were quantified according to Lichtenthaler (1987).

H_2_O_2_ was quantified as described in Alexieva et al. (2001). Fresh samples were homogenized in 0.1% (w/v) TCA, centrifuged, and the supernatant was reacted with 100 mM potassium phosphate buffer (pH 7.5) and 1 M KI. Absorbance was measured at 390 nm.

Malondialdehyde (MDA) content was determined using the TBA assay according to Heath and Packer (1968). Fresh tissue was homogenized in 0.1% (w/v) TCA with 20% (w/v) PVPP and centrifuged at 12,000 *g*. An aliquot of the supernatant was mixed with TCA (20%) and TBA (0.5%), incubated at 95°C for 30 min, cooled on ice, and centrifuged again. Absorbance was measured at 535 and 600 nm, and MDA content was expressed as nmol g^−1^ fresh weight.

### 2.5 Antioxidant Enzyme Activities

Root and leaf samples were homogenized in 100 mM potassium phosphate buffer (pH 7.5) containing 1 mM EDTA, 3 mM DTT, and 4% (w/v) PVPP (Gomes‐Junior et al. 2007). Extracts were centrifuged at 12,000 *g* for 30 min at 4°C, and protein content was determined according to Bradford (1976). Catalase (CAT, EC 1.11.1.6) and ascorbate peroxidase (APX, EC 1.11.1.11) activities were determined according to Kraus et al. (1995) and Moldes et al. (2008), respectively, with appropriate modifications. Absorbances were measured at 240 nm (CAT) and 290 nm (APX).

### 2.6 Gene Expression Analysis

Total RNA was extracted from root tissue using TRIzol Reagent (Invitrogen) following the manufacturer's protocol and methodological guidelines of Chomczynski and Sacchi (1987). Genomic DNA was removed using DNase I (Invitrogen). cDNA was synthesized with MultiScribe Reverse Transcriptase (Invitrogen).

qPCR was performed using a StepOne System (Applied Biosystems) and GoTaq qPCR Master Mix (Promega). For this experiment, the targeted genes were *IRON‐REGULATED TRANSPORTER 1* (*SlIRT 1*; locus number Solyc02g069200) (F: GAAATGGCTGTGGCTGGAAATC; R: CTGTAGGATGCAACCACCAAGG; 261 bp), *FERRIC REDUCTASE OXIDASE* (*SlFRO1*; locus number Solyc01g094910) (F: GTTCGTTTAGTCTCTGCTCGTG; R: TGAAGTGATGGTAAAAGGATGC; 153 bp), and *CLATHRIN ADAPTOR COMPLEXES SUBUNIT* (*CAC*; locus number Solyc08g006960) (F: CCTCCGTTGTGATGTAACTGG; R: ATTGGTGGAAAGTAACATCATCG; 173 bp), with the latter used as housekeeping gene. Primers were designed using Primer 3 (http://primer3plus.com/). Relative gene expression was determined according to Expósito‐Rodríguez et al. (2008) and Morohashi and Grotewold (2009).

### 2.7 Experimental Design and Statistical Procedures

The experiment followed a randomized block design with six genotypes, two nutrient treatments, and three replicates, each containing four plants. Data were subjected to ANOVA, and means were compared using Tukey's HSD test (*p* < 0.05). Statistical analyses in R language (version 4.5.0) were performed using RStudio IDE for Windows (version 2026.01.1).

## 3 Results

### 3.1 Morphological Characterization of Single and Double Hormonal Mutants Under +Fe and −Fe Conditions

The tomato *Never ripe* (*Nr*) mutant exhibited leaf and root morphology similar to WT, but its fruits showed incomplete ripening, remaining yellowish and firm. The *diageotropic* (*dgt*) mutant was characterized by hyponastic cotyledons and leaves, as well as a reduced number of lateral roots. In contrast, the *epinastic* (*epi*) mutant displayed epinastic leaves, thicker roots in general, an increased number of lateral roots, greater stem diameter, and fruits with a slightly yet distinctive pointed stylar tip (Figure 1).

**FIGURE 1:**
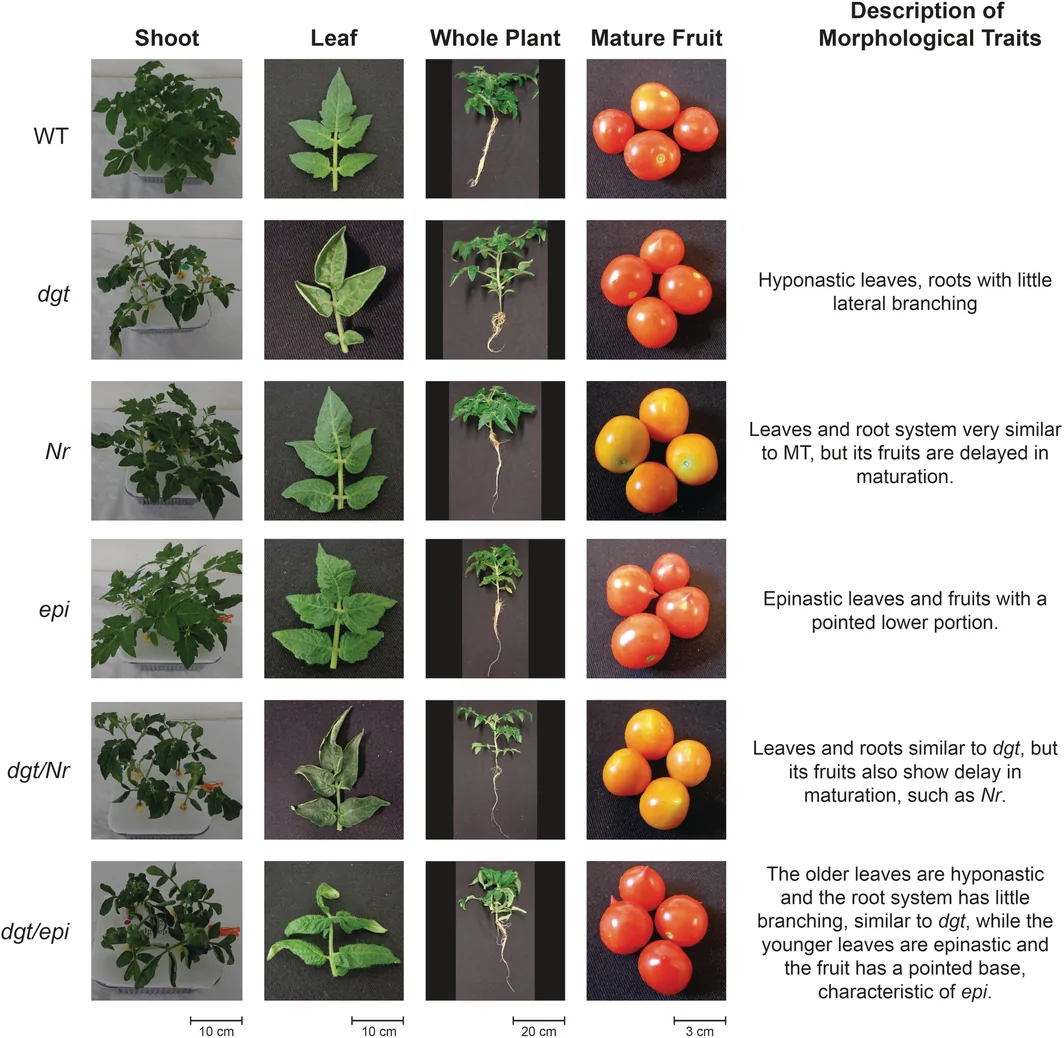
Morphological traits of tomato hormonal single mutants *diageotropica* (dgt), *Never ripe* (Nr), *epinastic* (epi), and double mutants *dgt/Nr* and *dgt/epi* compared to wild‐type (WT, cv. Micro‐Tom background).

The analysis of the double mutants revealed distinct phenotypic dominance patterns for *dgt/Nr* and *dgt/epi* mutants. The *dgt* allele exerted a predominant influence over *Nr* in vegetative structures, as the double mutant presented hyponastic leaves and reduced lateral root formation, similar to *dgt*. However, regarding fruit development, *dgt/Nr* displayed the blocked ripening phenotype, a characteristic trait of the *Nr* mutant, suggesting that the ethylene‐dependent ripening defect associated with the *Nr* allele overrides the *dgt* phenotype.

In *dgt/epi*, both alleles seem to exert organ‐ and stage‐dependent phenotypic interactions, as young leaves presented the *epi*‐like epinastic phenotype, whereas older leaves were hyponastic, suggesting a *dgt* influence. The root morphology indicated predominant effects of *dgt* over *epi*, as the double mutant exhibited reduced lateral root formation. Regarding fruit morphology, *dgt/epi* fruits phenocopied *epi* by retaining the distinctively pointed stylar tip, indicating an influential role for the *epi* allele in determining stylar apex shape.

### 3.2 PCA Analysis Reveals Genotype‐Dependent Responses to Fe Availability

To check overall relationships between genotypes, Fe availability, and morphophysiological variables, a Principal Component Analysis (PCA) was performed (Figure 2). The first two principal components (PCs) together explained 53.94% of the total data variance. The biplot revealed a clear separation based on both Fe availability and *dgt*‐related effects, forming four distinct clusters: (i) WT, *epi* and *Nr* under +Fe conditions, (ii) WT, *epi* and *Nr* under −Fe, (iii) *dgt*, *dgt/Nr* and *dgt/epi* under +Fe conditions, and (iv) *dgt*, *dgt/Nr* and *dgt/epi* under −Fe.

**FIGURE 2:**
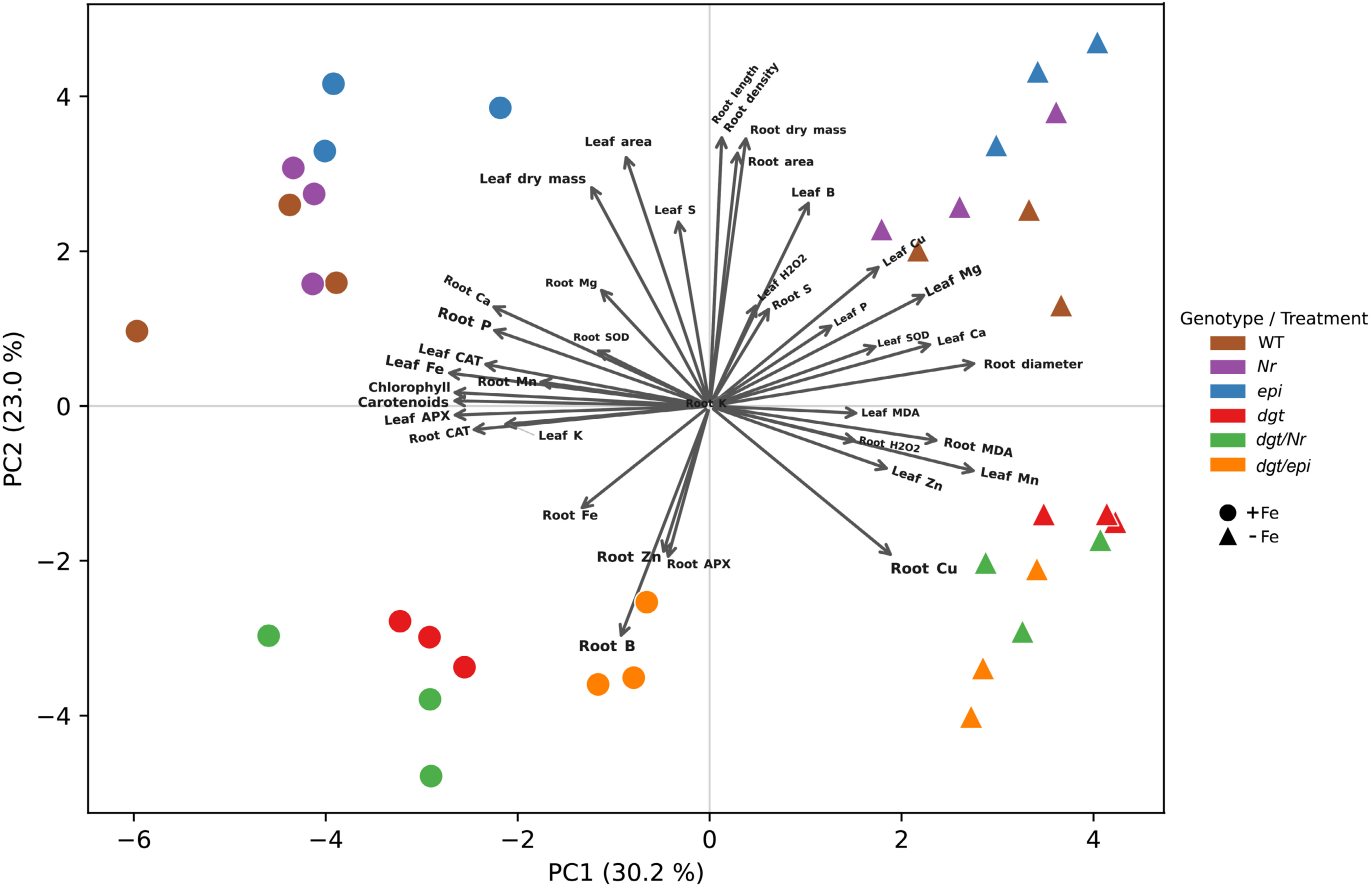
Principal component analysis (PCA) of morphological, biochemical, and nutritional traits in the tomato cv. Micro‐Tom background. Morphological variables include root length, density, area, and dry mass, root diameter, and leaf area and dry mass. Biochemical traits comprise root and leaf APX, CAT, MDA, and H_2_O_2_ contents, alongside leaf chlorophyll and carotenoid levels. Nutritional traits encompass root and leaf macronutrients (K, Ca, S, P, Mg) and micronutrients (B, Cu, Fe, Mn, Zn). Plants evaluated include the wild‐type (WT), hormonal single mutants (*diageotropica*, dgt; *Never ripe*, Nr; *epinastic*, epi), and double mutants (*dgt*/*Nr* and *dgt*/*epi*), cultivated under nutrient‐sufficient (+Fe) or Fe‐deficient (−Fe) solutions. Data were standardized.

PC1, which explained 31.82% of the variation, separated genotypes according to Fe treatment. +Fe was associated with higher pigment accumulation, catalase and APX activities, and increased Ca and P concentrations in the roots, whereas −Fe showed opposite loadings. −Fe was also associated with increased levels of oxidative stress markers (H_2_O_2_ and MDA), and higher Mn, Mg, and Ca accumulation in leaves, as well as Cu accumulation in roots.

PC2 explained 22.12% of the total variance in the dataset and separated the genotypes based on the influence of *dgt* allele. WT, *Nr*, and *epi* clustered together and were associated with higher accumulation of total biomass, larger leaf and root areas, and increased concentrations of B and S in leaves. Conversely, *dgt*, *dgt/Nr*, and *dgt/epi* genotypes showed negative loadings for these traits and were associated with higher APX activity and increased B, Zn, and Cu accumulation in roots.

Overall, the PCA results indicate that the *dgt* allele exerts predominant phenotypic effects over *Nr* and *epi* backgrounds, influencing biomass allocation, antioxidant defense mechanisms, and nutrient profile. In addition, the interaction between auxin‐ and ethylene‐hormonal mutants and Fe availability appears to be a key determinant of the morphophysiological responses in tomato, as evidenced by the formation of four well‐defined clusters in the PCA biplot.

### 3.3 Morphological Traits and Biomass Partitioning Under Fe Deficiency

In general, the low auxin‐sensitive *dgt* single mutant and the double mutants *dgt/Nr* and *dgt/epi* exhibited lower leaf (Figure 3A) and root areas (Figure 3B) than the values observed for WT, *Nr*, and *epi*. A similar pattern was observed for leaf and root dry biomass (Figure 3C,D). These differences between groups of genotypes were observed under both +Fe and −Fe conditions, suggesting a strong influence of the *dgt* allele on growth‐related traits (Table S1).

**FIGURE 3:**
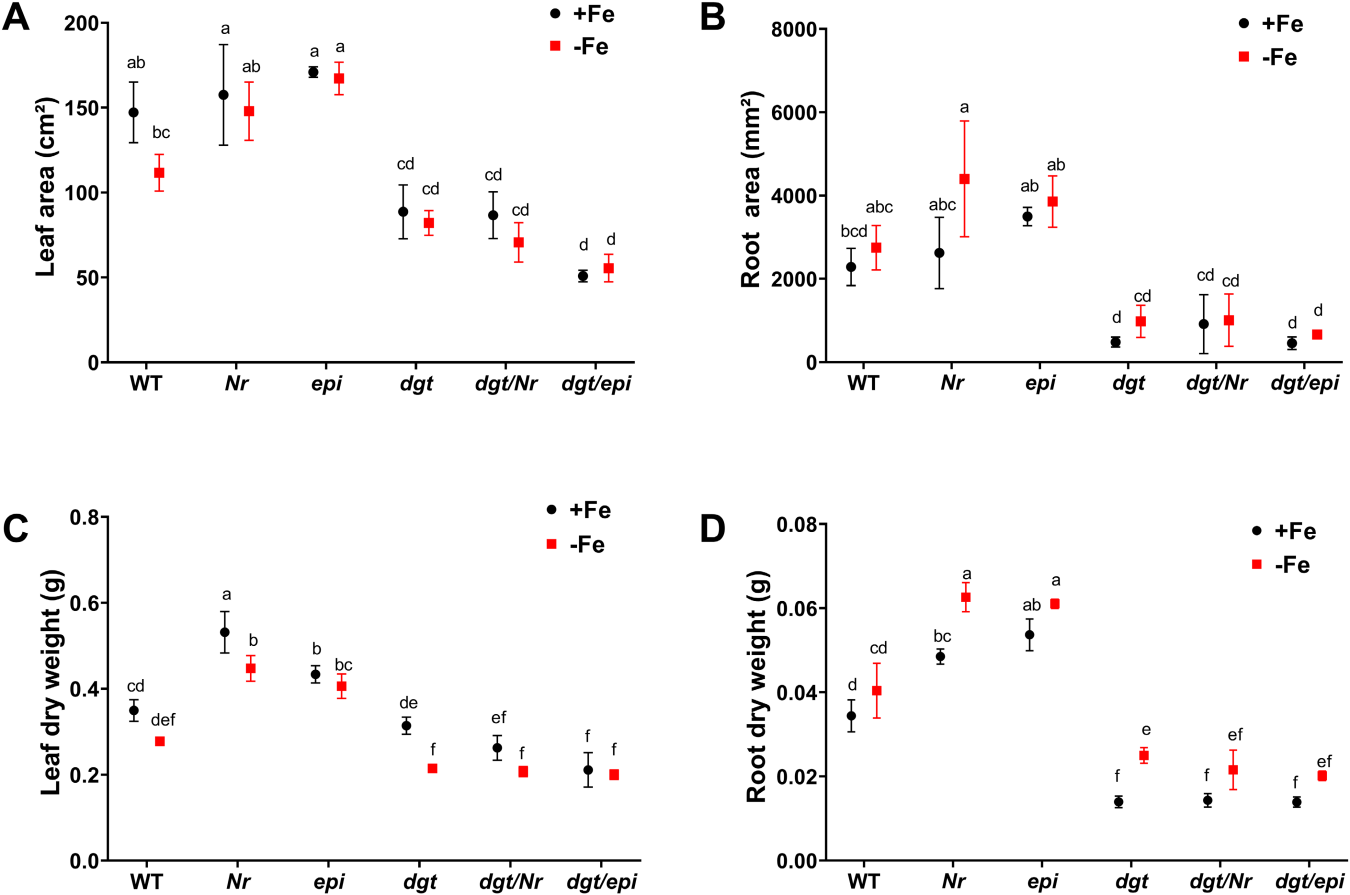
Plant growth parameters of wild‐type tomato cv. Micro‐Tom (WT), hormonal single mutants *diageotropica* (dgt), *Never ripe* (Nr), *epinastic* (epi), and double mutants *dgt/Nr* and *dgt/epi* cultivated under nutrient‐sufficient (+Fe) and Fe‐deficient (−Fe) solutions. (A) Leaf area; (B) root area; (C) leaf dry biomass; (D) root dry biomass. Values are mean ± SD (*n* = 3). Distinct letters indicate significant statistical differences between genotypes × treatments (Two‐way ANOVA followed by Tukey HSD test, *α* = 0.05).

Under +Fe conditions, *Nr* and *epi* accumulated approximately twice as much total dry biomass compared to WT and *dgt* single and double mutants. However, −Fe altered biomass allocation patterns in *Nr* and *dgt* mutants, which displayed reduced leaf biomass accompanied by a proportionally increased root biomass.

### 3.4 Photosynthetic Pigments and Differential Oxidative Stress Responses to Fe Availability Mediated by Auxin and Ethylene

Under control conditions, chloroplast pigment contents in *epi*, *dgt*, and *dgt/epi* were 27.8% lower than in WT (Figure 4A,B). Under −Fe conditions, chlorophyll and carotenoid levels became similar among genotypes, as WT, *dgt*, and *dgt/Nr* showed significant decreases under −Fe.

**FIGURE 4:**
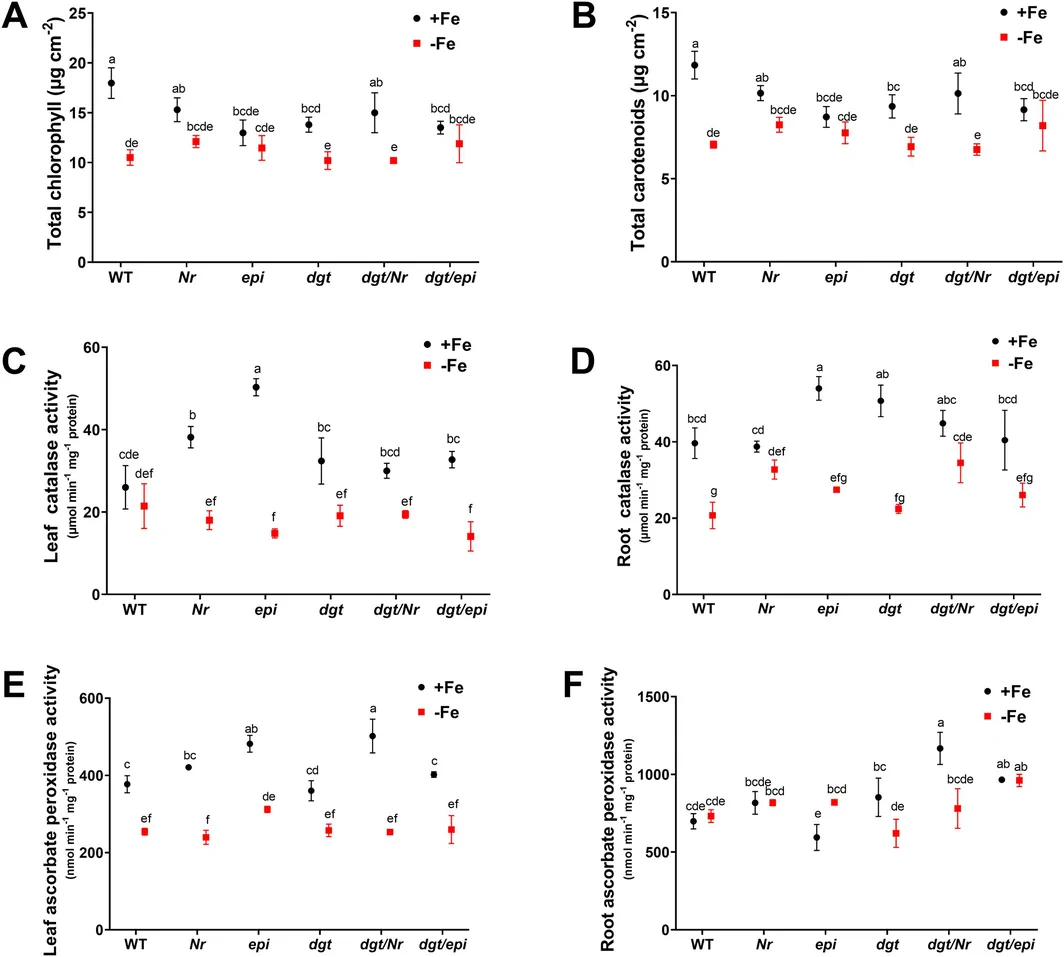
Chloroplast pigment levels and antioxidant enzyme activities of wild‐type tomato cv. Micro‐Tom (WT), hormonal single mutants *diageotropica* (dgt), *Never ripe* (Nr), *epinastic* (epi), and double mutants *dgt/Nr* and *dgt/epi* cultivated under nutrient‐sufficient (+Fe) and Fe‐deficient (−Fe) solutions. (A) Total chlorophyll; (B) total carotenoids; (C) leaf catalase activity; (D) root catalase activity; (E) leaf ascorbate peroxidase activity; (F) root ascorbate peroxidase activity. Values are mean ± SD (*n* = 3). Distinct letters indicate significant statistical differences between genotypes × treatments (Two‐way ANOVA followed by Tukey HSD test, *α* = 0.05).

Overall, H_2_O_2_ and malondialdehyde (MDA) levels were comparable among genotypes under +Fe, although *Nr* exhibited higher H_2_O_2_ concentration in leaves and *dgt/Nr* in roots. Under −Fe, WT showed increased H_2_O_2_ content in both leaves and roots, about 39% and 184.5% relative to control conditions, respectively, as well as increased leaf MDA content (50%), whereas mutant responses were less consistent. Notably, increased root H_2_O_2_ content was observed in *dgt* under −Fe (240%) (Table 1).

**TABLE 1:** Hydrogen peroxide (H_2_O_2_) and malondialdehyde (MDA) contents in leaves and roots of wild‐type tomato cv. Micro‐Tom (WT), hormonal single mutants (*diageotropica*, *dgt*; *Never ripe*, *Nr*; *epinastic*, *epi*), and double mutants (*dgt*/*Nr* and *dgt*/*epi*) cultivated under nutrient‐sufficient (+Fe) and Fe‐deficient (−Fe) solutions.

Genotype	Treatment	Leaf H_2_O_2_	Root H_2_O_2_	Leaf MDA	Root MDA
		nmol g^−1^ FW	nmol g^−1^ FW	nmol g^−1^ FW	nmol g^−1^ FW
WT	+Fe	141.51 ± 20.03 cdef	56.89 ± 6.35 bc	18.80 ± 0.55 c	4.66 ± 0.49 cde
WT	−Fe	196.34 ± 12.46 ab	161.62 ± 14.39 a	28.32 ± 6.68 ab	5.89 ± 0.79 abc
*Nr*	+Fe	218.68 ± 8.97 a	53.42 ± 10.06 bc	22.49 ± 0.53 abc	4.28 ± 0.61 de
*Nr*	−Fe	225.93 ± 12.91 a	72.84 ± 8.91 bc	25.54 ± 3.54 abc	6.15 ± 0.34 abc
*epi*	+Fe	117.42 ± 24.13 def	76.49 ± 20.37 bc	20.80 ± 2.50 bc	3.28 ± 0.52 e
*epi*	−Fe	143.51 ± 16.62 cdef	83.98 ± 19.29 b	21.38 ± 3.67 abc	6.79 ± 0.80 a
*dgt*	+Fe	143.99 ± 9.17 cde	42.14 ± 4.19 c	24.89 ± 1.45 abc	4.56 ± 0.60 cde
*dgt*	−Fe	183.74 ± 22.35 abc	143.86 ± 11.18 a	30.03 ± 4.41 a	5.55 ± 0.42 abcd
*dgt/Nr*	+Fe	149.02 ± 14.53 cd	135.77 ± 10.25 a	18.76 ± 1.41 c	5.08 ± 0.63 bcd
*dgt/Nr*	−Fe	164.81 ± 5.17 bc	132.99 ± 24.81 a	23.38 ± 0.83 abc	6.59 ± 0.26 ab
*dgt/epi*	+Fe	99.89 ± 8.50 f	74.82 ± 2.55 bc	25.44 ± 2.04 abc	5.48 ± 0.22 abcd
*dgt/epi*	−Fe	101.08 ± 10.60 ef	63.54 ± 2.29 bc	20.69 ± 3.02 bc	5.78 ± 0.45 abcd

*Note:* Values are mean ± SD (*n* = 3). Different letters indicate significant statistical differences for the genotype × treatment interaction (Two‐way ANOVA followed by Tukey's HSD test, *α* = 0.05).

Under −Fe conditions, root CAT activity was higher in Nr than in WT, whereas *Nr* did not differ clearly from its +Fe condition (Figure 4D). In contrast, root APX activity increased in *epi* under −Fe compared with the +Fe and reached values statistically similar to those observed in WT and Nr under −Fe (Figure 4F). Root H_2_O_2_ content did not significantly increase in either *Nr* or *epi* under −Fe, whereas root MDA content increased in both mutants, 43.7% in *Nr* and 107% in *epi* (Table 1). No additional antioxidant enzyme activities were evaluated in this study.

Taken together, these results indicate that Fe deficiency triggered genotype‐ and organ‐dependent changes in oxidative metabolism, as shown by increased root H_2_O_2_ in *dgt*, increased root MDA in *Nr* and *epi*, and distinct root CAT and APX activity patterns among the ethylene‐related mutants.

### 3.5 Nutritional Rearrangements and Ethylene‐Driven Regulation of Fe Acquisition Genes

The most striking effect of −Fe on the tomato nutrient profile was an increased accumulation of Cu in the roots of the low auxin‐sensitive *dgt*, *dgt/Nr*, and *dgt/epi* mutants, which exhibited nearly a twofold increase relative to the control conditions. In leaves, all genotypes displayed a 2‐ to 3‐fold increase in Mn content when grown under −Fe conditions (Table 2).

**TABLE 2:** Nutrient profile of leaves and roots of wild‐type tomato cv. Micro‐Tom (WT), hormonal single mutants (*diageotropica*, *dgt*; *Never ripe*, *Nr*; *epinastic*, *epi*), and double mutants (*dgt*/*Nr* and *dgt*/*epi*) cultivated under nutrient‐sufficient (+Fe) and Fe‐deficient (−Fe) solutions.

Organ	Genotype	Treatment	K	Ca	S	P	Mg	B	Cu	Fe	Mn	Zn
			mg g^−1^	mg g^−1^	mg g^−1^	mg g^−1^	mg g^−1^	μg g^−1^	μg g^−1^	μg g^−1^	μg g^−1^	μg g^−1^
Root	WT	+Fe	41.83 ± 9.58 a	81.88 ± 25.77 a	8.00 ± 3.87 a	41.14 ± 13.04 a	5.76 ± 1.91 a	113.70 ± 10.87 cdef	8.96 ± 1.75 d	174.08 ± 47.88 b	2534.02 ± 426.72 a	110.35 ± 39.42 a
Root	WT	−Fe	51.30 ± 4.64 a	13.93 ± 4.95 b	7.23 ± 0.51 a	11.41 ± 1.44 b	4.97 ± 2.75 a	95.22 ± 19.05 def	22.84 ± 2.35 cd	98.75 ± 18.77 b	997.85 ± 325.72 bc	68.27 ± 16.71 a
Root	*Nr*	+Fe	37.66 ± 2.23 a	84.60 ± 17.16 a	4.73 ± 1.37 a	45.20 ± 8.61 a	4.32 ± 1.63 a	137.41 ± 7.32 bcdef	9.62 ± 2.93 d	174.44 ± 54.16 b	2438.71 ± 297.86 ab	148.52 ± 34.11 a
Root	*Nr*	−Fe	58.41 ± 8.96 a	8.72 ± 1.72 b	5.71 ± 1.04 a	10.30 ± 0.57 b	2.73 ± 0.53 a	99.12 ± 12.53 cdef	32.56 ± 6.66 cd	122.97 ± 20.15 b	1415.40 ± 104.44 abc	92.24 ± 46.92 a
Root	*epi*	+Fe	44.84 ± 4.36 a	34.43 ± 12.67	6.44 ± 1.52 a	16.37 ± 7.31 b	5.57 ± 2.58 a	86.61 ± 8.06 f	10.69 ± 2.14 d	249.58 ± 145.94 b	1200.07 ± 490.11 abc	63.25 ± 11.85 a
Root	*epi*	−Fe	48.23 ± 13.15 a	11.11 ± 2.93 b	6.66 ± 1.80 a	10.42 ± 3.61 b	3.79 ± 1.80 a	88.01 ± 10.24 ef	28.32 ± 11.71 cd	157.74 ± 70.69 b	1102.17 ± 437.70 abc	80.84 ± 43.33 a
Root	*dgt*	+Fe	45.40 ± 2.44 a	13.87 ± 9.26 b	4.16 ± 0.81 a	16.38 ± 2.05 b	3.97 ± 0.65 a	185.16 ± 20.85 ab	21.90 ± 13.18 cd	580.10 ± 228.77 a	1898.67 ± 901.89 abc	124.34 ± 82.96 a
Root	*dgt*	−Fe	50.54 ± 7.09 a	8.94 ± 1.95 b	4.51 ± 1.00 a	9.61 ± 1.87 b	3.00 ± 0.30 a	136.68 ± 37.49 bcdef	57.94 ± 18.87 bc	104.28 ± 19.67 b	1066.21 ± 309.44 abc	102.46 ± 46.97 a
Root	*dgt/Nr*	+Fe	43.80 ± 1.29 a	25.48 ± 33.24 b	4.03 ± 0.25 a	18.69 ± 15.88 b	2.89 ± 0.60 a	214.08 ± 2.41 a	23.89 ± 11.05 cd	215.91 ± 101.15 b	1537.50 ± 512.39 abc	134.82 ± 67.75 a
Root	*dgt/Nr*	−Fe	44.92 ± 0.30 a	7.76 ± 2.58 b	7.26 ± 1.29 a	10.47 ± 2.12 b	2.78 ± 0.50 a	150.03 ± 26.85 bcd	75.21 ± 27.40 ab	110.91 ± 56.74 b	1777.18 ± 1070.60 abc	130.14 ± 39.18 a
Root	*dgt/epi*	+Fe	41.56 ± 6.48 a	7.60 ± 1.47 b	4.08 ± 0.68 a	8.27 ± 2.21 b	3.57 ± 0.77 a	153.64 ± 31.91 bc	46.08 ± 21.91 bcd	376.79 ± 111.01 ab	1055.76 ± 257.12 abc	204.72 ± 76.09 a
Root	*dgt/epi*	−Fe	40.89 ± 1.03 a	7.37 ± 3.76 b	6.84 ± 0.43 a	9.16 ± 0.79 b	2.15 ± 0.30 a	144.30 ± 5.38 bcde	107.22 ± 18.97 a	209.82 ± 42.36 b	907.77 ± 135.60 c	174.63 ± 51.10 a
Leaf	WT	+Fe	57.47 ± 9.42 ab	41.72 ± 6.19 b	13.96 ± 1.52 abc	10.69 ± 1.95 bc	10.95 ± 1.05 bc	159.72 ± 20.63 ab	12.36 ± 0.05 bcd	99.73 ± 3.55 cde	128.94 ± 21.72 gh	17.84 ± 3.47 c
Leaf	WT	−Fe	43.76 ± 3.49 bc	51.54 ± 4.05 ab	13.16 ± 1.19 abc	11.41 ± 1.47 bc	13.11 ± 0.31 abc	190.78 ± 40.68 ab	14.50 ± 1.64 ab	68.34 ± 8.03 abc	261.60 ± 33.14 de	19.01 ± 4.04 bc
Leaf	*Nr*	+Fe	53.36 ± 3.15 abc	41.44 ± 5.94 b	15.03 ± 1.91 abc	11.25 ± 0.34 bc	11.85 ± 1.45 abc	175.22 ± 14.66 ab	13.51 ± 0.25 abc	103.49 ± 8.38 ab	160.00 ± 41.33 fgh	21.80 ± 1.38 abc
Leaf	*Nr*	−Fe	40.52 ± 5.15 c	52.93 ± 5.07 ab	14.18 ± 1.50 abc	12.95 ± 2.03 abc	13.41 ± 1.27 ab	183.58 ± 64.13 ab	14.90 ± 1.42 ab	71.17 ± 11.50 bcde	308.78 ± 50.16 cd	28.02 ± 1.62 a
Leaf	*epi*	+Fe	60.13 ± 9.52 a	45.56 ± 3.53 ab	19.10 ± 0.20 a	13.67 ± 0.50 ab	11.05 ± 0.01 abc	199.90 ± 42.33 ab	14.52 ± 1.24 ab	122.53 ± 16.77 a	103.69 ± 31.85 h	18.77 ± 3.28 bc
Leaf	*epi*	−Fe	47.88 ± 1.46 abc	61.79 ± 5.25 a	18.64 ± 2.74 abc	16.41 ± 0.35 a	14.36 ± 0.87 a	230.22 ± 29.53 a	16.75 ± 0.68 a	64.34 ± 9.05 de	265.29 ± 47.46 de	24.97 ± 4.15 abc
Leaf	*dgt*	+Fe	53.63 ± 3.76 abc	45.38 ± 3.11 ab	13.19 ± 1.25 abc	11.21 ± 1.62 bc	11.24 ± 0.53 abc	111.51 ± 38.41 b	9.54 ± 0.46 d	94.99 ± 22.28 abcde	203.74 ± 15.57 ef	21.60 ± 0.33 abc
Leaf	*dgt*	−Fe	42.44 ± 3.28 bc	56.21 ± 5.83 b	12.52 ± 1.15 bc	12.91 ± 0.52 abc	13.39 ± 0.82 ab	185.44 ± 27.68 ab	13.61 ± 1.19 abc	71.52 ± 2.68 bcde	443.84 ± 16.11 a	26.23 ± 1.40 ab
Leaf	*dgt/Nr*	+Fe	51.44 ± 2.54 abc	40.55 ± 6.82 ab	11.11 ± 3.99 c	9.01 ± 2.35 c	9.89 ± 1.99 c	115.63 ± 28.07 b	10.69 ± 1.60 cd	97.17 ± 17.72 abcd	165.91 ± 52.39 fgh	19.52 ± 1.91 bc
Leaf	*dgt/Nr*	−Fe	44.05 ± 2.08 bc	56.01 ± 1.73 ab	12.45 ± 0.66 bc	11.40 ± 0.89 bc	13.09 ± 0.53 abc	166.15 ± 20.77 ab	13.88 ± 0.69 abc	62.04 ± 1.28 e	386.32 ± 39.82 ab	25.84 ± 0.70 abc
Leaf	*dgt/epi*	+Fe	56.84 ± 4.26 ab	49.16 ± 9.20 ab	15.09 ± 1.75 abc	14.69 ± 0.72 ab	11.28 ± 1.00 abc	128.05 ± 16.10 b	14.03 ± 2.35 abc	97.21 ± 4.04 abcd	180.66 ± 38.98 fg	24.31 ± 4.25 abc
Leaf	*dgt/epi*	−Fe	53.46 ± 6.25 abc	49.58 ± 10.57 ab	12.24 ± 3.85 c	14.29 ± 3.12 ab	12.30 ± 1.89 abc	138.91 ± 33.91 ab	15.09 ± 0.77 ab	63.03 ± 6.04 e	359.03 ± 50.75 bc	29.11 ± 2.47 a

*Note:* Values are mean ± SD (*n* = 3). Different letters indicate significant statistical differences for the genotype × treatment interaction (Two‐way ANOVA followed by Tukey's HSD test, *α* = 0.05).

*IRON REGULATED TRANSPORTER 1* (*IRT1*) transcript levels in the roots of *dgt/epi* mutant were 1.81 log_2_FC higher than WT under +Fe conditions. −Fe led to increased *IRT1* levels only in WT, *epi*, and *dgt/epi* mutants (Figure 5A; Table S1).

**FIGURE 5:**
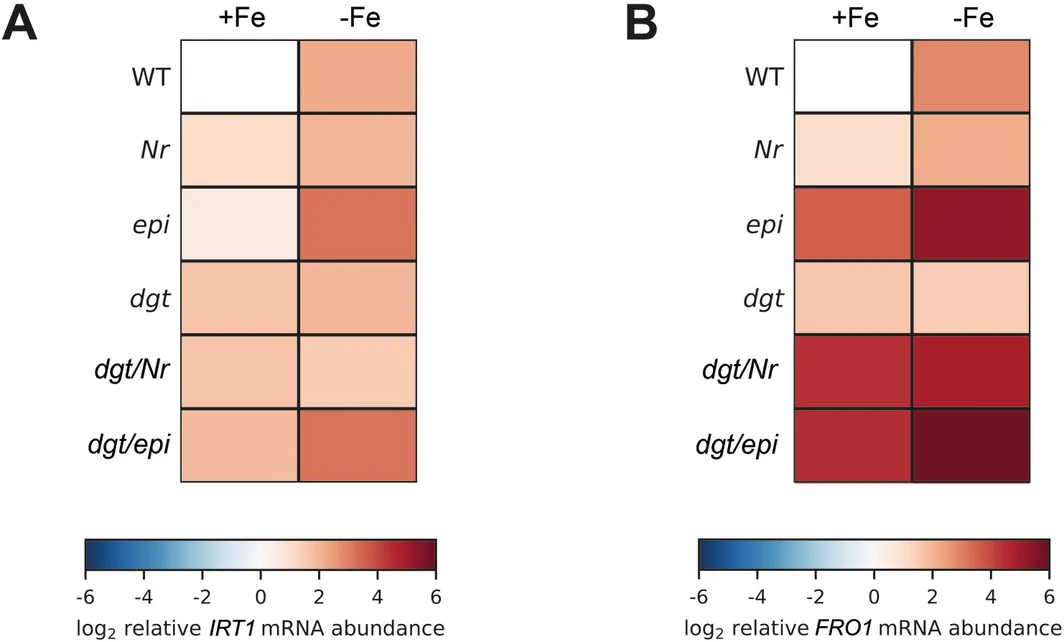
Heatmap representation of log_2_ root mRNA abundance of Fe‐related genes (A) *IRON REGULATED TRANSPORTER 1* (*IRT1*) and (B) *FERRIC REDUCTASE OXIDASE 1* (*FRO1*), normalized against wild‐type (WT) under nutrient‐sufficient conditions (+Fe). The color scale represents log_2_‐fold change (log_2_FC) values, where red indicates up‐regulation and blue indicates down‐regulation. Values represent means (*n* = 3). Statistical differences between genotypes × treatments (Two‐way ANOVA followed by Tukey HSD test, *α* = 0.05) are provided in Table S1.

mRNA levels of *FERRIC REDUCTASE OXIDASE 1* (*FRO1*), which encodes an important ferric chelate reductase, were markedly up‐regulated in the *epi* single and *dgt/epi* double mutant, reaching log_2_‐fold changes (log_2_FC) of 3.58 and 4.43, respectively. An additive effect was observed in the *dgt/Nr* double mutant, which showed a log_2_FC increase of 4.45 in *FRO1* transcript levels, whereas neither the *Nr* nor the *dgt* single mutants displayed significant *FRO1* up‐regulation. Under −Fe conditions, *FRO1* transcription was even further enhanced in *epi* and *dgt/epi* mutants, by up to 1.58 log_2_FC relative to Fe‐sufficient conditions (Figure 5B; Table S1).

Collectively, these results suggest that enhanced ethylene biosynthesis associated with the *epi* mutation promotes higher expression of Fe‐responsive genes and a heightened transcriptional response to −Fe in tomato roots. Our findings further suggest that ethylene may play a more prominent regulatory role than auxin in the transcriptional control of Fe homeostasis.

## 4 Discussion

Fe availability in soils is highly heterogeneous; thus, the ability of plants to adjust morphophysiological and metabolic processes under −Fe is critical for both agronomic performance and ecological fitness. Our results demonstrate that −Fe responses in tomato are shaped by a complex interaction between auxin and ethylene signaling, superimposed on strong Fe‐driven metabolic constraints, particularly those related to oxidative metabolism.

### 4.1 Influence of Ethylene Over Auxin in the Transcriptional Control of Fe Acquisition

Fe deficiency is primarily sensed by roots, triggering transcriptional, biochemical, and morphological responses aimed at improving Fe mobilization and uptake (Lucena et al. 2015; Liang 2022). *S. lycopersicum* relies on a reduction‐based strategy (Strategy I), involving ferric chelate reduction by FRO1 and subsequent Fe^2+^ uptake into root cells via IRT1 (Connorton et al. 2017). Although auxin and ethylene are known to interact in the regulation of this strategy, our data indicate a strong role of ethylene in the transcriptional activation of Fe acquisition genes.

The strong induction of *FRO1* and *IRT1* transcripts in the *epi* and *dgt/epi* mutants under both +Fe and −Fe conditions contrasts with the lack of induction observed in the auxin‐insensitive *dgt* mutant (Figure 5). These results indicate that enhanced ethylene biosynthesis is sufficient to trigger Fe acquisition gene expression even in the presence of *dgt*‐dependent impaired auxin sensitivity. This suggests that ethylene acts either downstream of or independently of auxin in this regulatory pathway. This interpretation aligns with studies showing that ethylene positively regulates ferric reductase activity and Fe‐uptake responses (Lucena et al. 2006, 2015), whereas the disruption of auxin signaling alone is insufficient to sustain full transcriptional activation of Fe‐responsive genes.

The auxin‐ethylene pattern observed in tomato is consistent with regulatory modules described in other Fe acquisition systems, although important species‐specific differences must be considered. As a Strategy I species, tomato shares with *Arabidopsis thaliana* a reduction‐based mechanism in which rhizosphere acidification, ferric reduction by FRO proteins, and Fe^2+^ uptake by IRT transporters are central components of the Fe‐deficiency response (Kobayashi and Nishizawa 2012; Hindt and Guerinot 2012). In *Arabidopsis*, ethylene positively regulates Fe‐deficiency responses, at least in part through the EIN3/EIL1‐dependent stabilization of FIT (Lingam et al. 2011), whereas auxin contributes to root developmental remodeling and can promote FIT/FRO2 expression through NO‐dependent signaling (Chen et al. 2010). In agreement with this framework, our results showed that the enhanced ethylene response in *epi* and *dgt/epi* was associated with stronger *SlFRO1* and *SlIRT1* expression, whereas impaired auxin sensitivity in *dgt*‐containing genotypes mainly constrained root architecture.

Comparisons with rice require particular caution because *Oryza sativa* combines a Strategy II system based on deoxymugineic acid secretion and Fe(III)‐phytosiderophore uptake through specific transporters, with a Strategy I‐like capacity for Fe^2+^ uptake (Inoue et al. 2009; Lee et al. 2009; Kobayashi and Nishizawa 2012). Nevertheless, studies in rice also indicate that ethylene can stimulate Fe acquisition genes through OsIRO2‐dependent pathways and that auxin‐related factors influence root growth and Fe accumulation (Wu et al. 2011; Qi et al. 2012; Shen et al. 2015). Therefore, the tomato data suggest a conserved functional pattern in which ethylene is more directly associated with Fe acquisition signaling, while auxin primarily shapes root structure. However, differences in regulatory architecture among species prevent the conclusion that this pattern is universal across Strategy I and Strategy II plants.

### 4.2 Root and Shoot Development Are Constrained by Auxin Sensitivity Regardless of Fe Availability

Another notable effect of −Fe concerns alterations in the root architecture, particularly enhanced root branching and the formation of root hairs (Jin et al. 2008; Zhang et al. 2018). Furthermore, auxin is known to be a master controller of root development (Jan et al. 2024), interacting with ethylene to regulate lateral and adventitious roots in tomato (Negi et al. 2010). However, in contrast to the gene expression responses observed, root architecture and overall plant growth were severely constrained by *dgt*‐related auxin responsiveness. The *dgt* allele exerted clear predominant phenotypes over *Nr* and *epi* backgrounds, resulting in reduced lateral root formation, smaller root systems, and decreased leaf area regardless of Fe availability (Figure 3). Although −Fe induced modest increases in root biomass in some genotypes, neither blocked ethylene perception (*Nr*) nor enhanced ethylene biosynthesis (*epi*) was able to rescue the impaired root development imposed by the auxin insensitivity in *dgt*‐containing genotypes. These findings reinforce the role of auxin as a main regulator of root and leaf development and indicate that, while ethylene modulates Fe acquisition at the transcriptional level, auxin sets the structural framework within which −Fe responses operate.

### 4.3 Photosynthetic Pigments and Oxidative Metabolism Reflect Primary Fe‐Driven Constraints

The reduction in chlorophyll and carotenoid contents under −Fe across all genotypes reflects the well‐established requirement for Fe in chlorophyll biosynthesis and photosynthetic function, accounting for the convergence of pigment levels under −Fe and the typical chlorotic phenotype. This impairment of the photosynthetic apparatus is closely linked to oxidative stress, as −Fe compromises both electron transport processes and Fe‐dependent antioxidant enzymes (Vigani 2012). In leaves, CAT activity declined in response to −Fe rather than genotype (Figure 4C). As CAT is a heme‐containing Fe‐dependent enzyme, reduced Fe availability directly limits its synthesis and catalytic activity. APX showed a similar Fe‐dependent decline in leaves across all genotypes (Figure 4E).

However, antioxidant enzyme activities did not show a uniform Fe‐dependent decline in roots. CAT activity exhibited a genotype‐dependent pattern, with higher activity in *Nr* under −Fe than in WT roots under the same condition (Figure 4D). Root APX activity varied among genotypes and increased in *epi* under −Fe compared with +Fe condition (Figure 4F). These root responses suggest that ethylene‐related mutations affected CAT and APX in different ways under Fe deficiency. The higher CAT activity in *Nr* roots may reflect a genotype‐specific reorganization of H_2_O_2_ detoxification when ethylene perception through the NR receptor is impaired, rather than a simple loss of antioxidant response. This interpretation is consistent with previous evidence that the NR receptor can modulate oxidative‐stress‐related biochemical pathways in a tissue‐ and stress‐dependent manner (Monteiro et al. 2011). Conversely, the increase in APX activity in *epi* is consistent with an association between exaggerated ethylene responsiveness and activation of the ascorbate‐dependent H_2_O_2_‐scavenging pathway. To our knowledge, the relationship between the exaggerated ethylene response of *epi* and APX activity in −Fe tomato roots has not been directly examined.

Since CAT and APX differ in their biochemical requirements and roles in H_2_O_2_ removal, these enzyme‐specific responses should not be interpreted as a uniform increase in overall antioxidant efficiency (Asada 1999; Mhamdi et al. 2012; Noctor et al. 2018). These enzymatic enhancements, however, were not accompanied by lower MDA levels in *Nr* or *epi* (Table 1), suggesting that ROS production, compartmentalization, substrate availability, or an imbalance among other antioxidant components may have exceeded the protection conferred by CAT or APX alone.

These results also suggest that, while the overall oxidative burden under −Fe is largely Fe‐driven, *Nr*‐dependent ethylene perception may contribute to alternative antioxidant adjustments in roots. Overall, the changes in H_2_O_2_ and MDA levels appear to be primarily driven by −Fe itself rather than by hormone‐related genetic backgrounds.

### 4.4 Altered Mineral Profiles Reflect Compensatory Responses to −Fe

Fe absence promoted pronounced changes in mineral nutrition, most notably a consistent increase in foliar Mn accumulation across all genotypes and a marked enhancement of Cu accumulation in roots, particularly in *dgt*‐based backgrounds. The generalized increase in foliar Mn content under −Fe is consistent with the reduced substrate specificity of high‐affinity Fe transporters, such as IRT1, which can mediate the uptake of other divalent cations under Fe‐limiting conditions (Korshunova et al. 1999; Rai et al. 2021). A comparable response occurs in rice, which compensates for low Fe uptake by activating the transcription factor *OsbHLH156‐OsIRO2*, inducing the accumulation of Cu, Mn, and Zn (Zhu et al. 2024).

In contrast, the nearly twofold increase in root Cu accumulation observed specifically in *dgt*, *dgt/Nr*, and *dgt/epi* mutants suggests that impaired auxin sensitivity may influence the extent or partitioning of metal uptake and retention in roots during Fe stress. The elevated levels of Cu, Mn, and Zn under −Fe may partially compensate for impaired Fe‐dependent antioxidant systems, as these metals act as cofactors for alternative ROS‐scavenging enzymes. This mechanism may explain the lack of a consistent increase in lipid peroxidation (MDA levels) in some genotypes despite reduced CAT and APX activities.

## 5 Conclusions

The employment of tomato hormonal double mutants represents an effective strategy for unraveling complex gene interactions and signaling pathways in plants. This study demonstrated that *Nr* and *epi*‐dependent ethylene signaling strongly contributes to the transcriptional regulation of Fe acquisition genes, acting as an influential driver for the upregulation of *FRO1* and *IRT1*, even when auxin sensitivity is impaired. Conversely, *dgt*‐related auxin sensitivity functions as the main regulator of plant morphology, exerting a dominant effect over *Nr‐* and *epi*‐dependent ethylene perception and biosynthesis to determine root development and architecture (Figure 6). These integrated responses improve our understanding of the biochemical mechanisms that enable plants to survive in −Fe conditions, such as the strategic accumulation of alternative metal cofactors (notably Mn and Cu) to control oxidative damage when Fe‐dependent antioxidant enzymes are compromised. Future research incorporating a broader range of mutants and combinations via cross−Fertilization will be crucial to explore additional signaling routes and the intricate crosstalk influencing nutrient homeostasis in plants.

**FIGURE 6:**
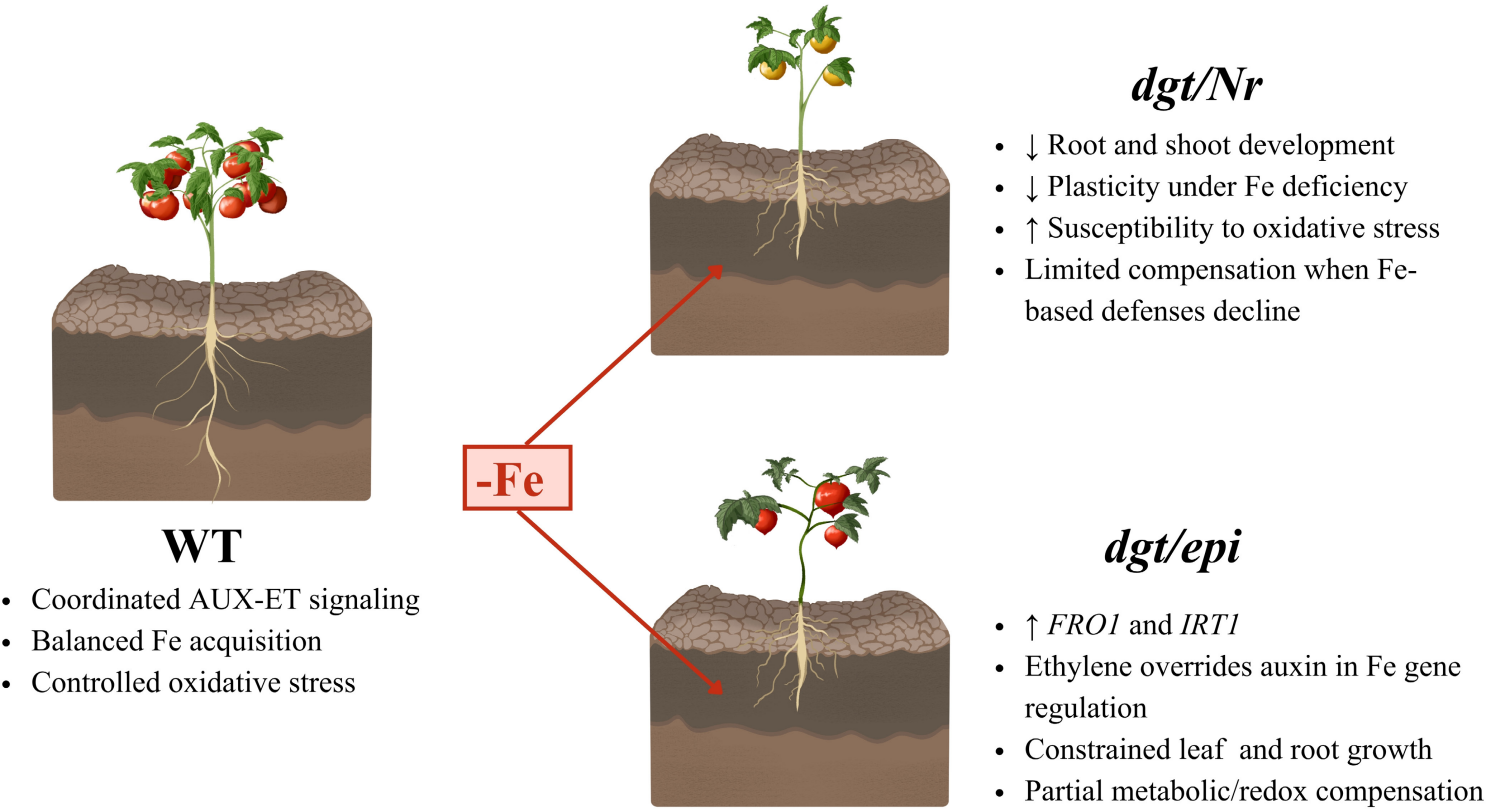
Comparative analysis of Fe deficiency (−Fe) responses between wild‐type tomato cv. Micro‐Tom (WT) and the hormonal double mutants *dgt/Nr* and *dgt/epi*.

## Author Contributions

C.C.M. and R.F.C. conceived and designed the research. C.C.M. performed the experiments and data collection. C.C.M., A.V.M., E.A.C.F., R.A.A., T.T., R.M.P., and F.R.R.A. curated and analyzed the data. A.V.M., F.R.R.A., and M.A.G. wrote the original draft. F.R.R.A. and R.F.C. revised the manuscript.

## Funding

This study was financed, in part, by the São Paulo Research Foundation (FAPESP), Brazil, #13/04316‐2.

## Supporting information


**Table S1:** Morphometric, biochemical and gene expression data of wild‐type tomato cv. Micro‐Tom (WT), hormonal single mutants (diageotropica, dgt; Never ripe, Nr; epinastic, epi), and double mutants (dgt/Nr and dgt/epi) cultivated under nutrient‐sufficient (+Fe) and Fe‐deficient (−Fe) solutions. Values are mean ± SD (*n* = 3). Different letters indicate significant statistical differences for the genotype × treatment interaction (Two‐way ANOVA followed by Tukey's HSD test, *α* = 0.05).

## Data Availability

The datasets generated and analyzed during the current study are available from the corresponding author upon reasonable request.
